# Adversity, attachment and emotion recognition in BPD adolescents: the distinct roles of disengaged and controlling environment

**DOI:** 10.1186/s40359-022-00788-7

**Published:** 2022-04-04

**Authors:** Marion Robin, Jean Belbèze, Alexandra Pham-Scottez, Mario Speranza, Gérard Shadili, Jerôme Silva, Maurice Corcos

**Affiliations:** 1 Department of Adolescent and Young Adult Psychiatry, Institut Mutualiste Montsouris, 42 boulevard Jourdan, 75014 Paris, France; 2 Paris Descartes University, Paris, France; 3 University Hospital Group Paris Psychiatry and Neurosciences, Paris, France; 4 Versailles General Hospital, Le Chesnay, France; 5 Paris-Saclay University, UVSQ, CESP, INSERM U1178, Team PsyDev, Gif-sur-Yvette, France

**Keywords:** Borderline personality disorders, Facial emotion recognition, Adversity, Disengaged environment, Controlling environment, Maltreatment, Attachment, Parent–child bonding

## Abstract

**Background:**

Literature data about emotion perception in patients with borderline personality disorders (BPD) revealed some discrepancies between some patients that are vigilant and accurate to detect their emotional environment and others that are impaired at identifying emotions of others. Even if some links between childhood adversity and facial affect recognition have been established, there is a need to understand the heterogeneous psychobiological mechanisms underlying this association. The aim is to distinguish in a BPD sample, the links between facial emotion recognition (FER) and adversity types (maltreatment and parental bonding), by evaluating two dimensions of disengaged and controlling environment.

**Method:**

The study includes BPD adolescents (n = 45) and healthy controls (HC, n = 44): two scores of disengaged environment (parental low care; emotional and physical neglect) and controlling environment (high level of parenting control; emotional, physical and sexual abuse) were established and correlated to FER, as well as to attachment dimensions. Multiple linear regression analyzes were conducted to evaluate the effect of disengaged and controlling dimensions, on FER scores of sensitivity and accuracy, including anxious and avoidant attachment as covariables.

**Results:**

Analyzes revealed that a disengaged environment was positively correlated to sensitivity in BPD patients, and the correlation was negative in the HC group. Controlling environment was negatively associated to accuracy of emotion in BPD. Avoidant and anxious attachment did not influence these associations.

**Conclusions:**

These results suggest that distinct adverse experiences account for the heterogeneity observed in emotion regulation in BPD patients.

## Background

Adults and adolescents with borderline personality disorders (BPD) are thought to be highly sensitive to external features of others, such as facial emotions, but they are at the same time described as impaired in identifying correctly these emotions and in inferring mental states of others [1]. The mixed results produced by studies on mentalization and facial emotion recognition (FER) included the description of lower, unchanged or enhanced capacities in patients with BPD compared to healthy controls (HC) [2–4]. A few factors have been envisaged (age, BPD severity or experimental parameters) to explain this discrepancy, but the mechanisms underlying emotion recognition in its diversity are still difficult to understand. Literature on BPD mentalizing revealed the role of two concepts, which may play a central role in variations of patients’ ability to perceive emotional states of others: childhood trauma and attachment [5, 6]. The role of childhood adverse events is recognized in the pathogenesis of BPD: severe maltreatment and parental dysfunction are dose-dependent risk factor in borderline features among children, adolescents and adult with BPD, leading to a harsher and earlier disorder [7]. Correlations have been made between severity of maltreatment and FER in BPD patients: hyper- and hyposensitivity to emotions have been envisaged alternatively as an attempt of BPD patients to search for early signs of environmental threat, or contrarily as a protective mentalizing inhibition when trauma is severe [2, 6, 8, 9]. It was also suggested that the nature of childhood trauma was a crucial factor in determining its influence. In this sense, effects of abuse or severe maltreatment have been described with regard to FER skills [5, 10], but the specific effects of parental nonavailability (e.g. low level of care or neglect) have been less explored. However, in the line of Bowlby’s clinical development [11], experiences of inconsistent support may lead some individuals to develop vigilant attitudes to others’ emotional signals and feeling of abandonment. This type of environment, alternatively described as disengaged parenting, hidden trauma or maternal withdrawal, has been associated with features of BPD, as well as with child caregiving attitudes and insecure attachment [12–14]. The links between attachment and FER in BPD have also been explored and the results suggest that individuals with high levels of attachment anxiety, because of their fear of rejection and abandonment, would develop hypersensitivity to external features of others, including affective facial expressions [15], whereas avoidant attachment is supposed to impede FER because of individuals’ tendency to deactivate attachment needs and concerns. However, experimental studies have yielded mixed results there again [9].

Given this complexity, it is unclear how adversity and attachment are related to FER outcomes, and there is strong needs to understand the source of a discrepancy in diverse if not opposite aspects of borderline emotion recognition. BPD can be observed among up to 50% of inpatients adolescents [16] and is associated to significant social dysfunction, high levels of suicide and comorbidities, which reinforces the importance of preventing this disorder [17]. The link between childhood adversity and poor mental health outcomes is now well established [18] and there is a recent expressed need to move beyond correlational research on the psychobiological mechanisms underlying these associations [19]. The aim of this research is to distinguish the links between borderline FER and adversity types (maltreatment and parental bonding), by evaluating two dimensions of disengaged and controlling environment. In line with previous studies and with Bowlby’s clinical observations, our first hypothesis was that each adversity dimension would be correlated with FER through its own effect, including enhancement in FER with disengaged environment and decreased FER with controlling environment. The second hypothesis was that the links between adversity and FER remain significant even when attachment variables are associated.

## Methods

The study population was composed of 45 BPD adolescents (87% of girls), with a mean age of 16.5 years (sd = 1.4), and 44 healthy matched controls. The sample was issued from a European network investigating BPD in adolescence (European Research Network on Borderline Personality Disorder, EURNET BPD [20]). The research network involved 5 psychiatric centers specialized in adolescents in France, Belgium, and Switzerland. Patients (15 to 19 years old) were screened following the DSM-IV criteria for BPD. Non-inclusion criteria were diagnosed schizophrenia and any chronic or life-threatening medical illness. A sample of healthy controls (HC) matched individually by gender, age, and socio-economic status was recruited by announcement in school facilities. They were excluded if they were positive for a DSM-IV diagnosis of personality disorder. A French ethics committee approved the study (authorization n◦ 0611259). After complete description of the study, parents and adolescents both provided their written inform consent prior to the experiment. In accordance with the legal status of the study, only participants in the control group received compensation for their participation. All subjects have been assessed for diagnoses of axis-I and axis-II disorders, and have completed a self-reported questionnaire with psychopathological data, including attachment, maltreatment and parental bonding, and performed the FER task. BPD diagnosis was verified after administration of the Structured Interview for DSM Personality disorders [21] (SIDP-IV). All BPD criteria measures range on a Likert-scale from 0 to 3.

Psychiatric comorbidity was explored using SIPD-IV for axis-II disorders, and for axis-I, a semi-structured interview assessing DSM-IV criteria was used (Kiddie-Schedule for Affective Disorders and Schizophrenia, Kiddie-SADS). Adolescents with a diagnosis of schizophrenia, with any chronic and (or) serious medical illness involving vital prognosis, and adolescents with a mental retardation, were excluded from the sample.

### Measures

#### Emotion recognition

Emotion recognition was assessed with a task that comprised 36 trials presented in random order, taken from the empirically valid and reliable pictures of facial affect series of Ekman and Friesen [22, 23]. Each trial began with a neutral face gradually morphed into one of the six-prototypical emotions—sadness, anger, happiness, disgust, surprise, and fear—according to forty 2.5% incremental stages (500 ms per picture, 20 s per trial).

**Fig. 1:**
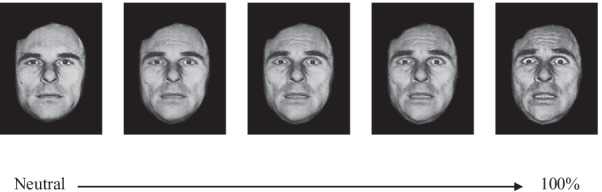
Stimulus example for the expression of fear

Participants were asked to watch the changes in facial expression and report the emotion expressed whenever they thought they had identified it. They could change their initial response at any time and as often as necessary by clicking again on one of the response buttons. At the end of each trial (40th stage), they had to indicate their final choice. With this paradigm, two FER skills were scored: accuracy was measured by the final success rate (i.e., percentage of correct response at 100% expression), and sensitivity by the precociousness of a right emotional recognition. It is measured by the difference between the final stage (40th stage) and the mean number of stages required for the accurate identification of facial emotions, across the trials in which the participants successfully recognized the final expression (Sensitivity = 40 − n). The greater is the difference between 40 and the number of images needed, the earlier the emotional recognition and the higher the sensitivity (Fig. 1).

#### Psychopathological measures

The retrospective assessment of childhood maltreatment was performed using the Childhood Trauma Questionnaire [24] (CTQ). It is a 28 items self-report instrument with five-point Likert-scale, exploring five types of maltreatment: Sexual abuse, Physical abuse, Physical neglect, Emotional abuse and Emotional neglect. Cut-off scores were used to define the presence of each type of trauma [25]. The Parental Bonding Instrument (PBI) is a self-administered questionnaire, which is widely used to measure the subjective experience of parent–child bonding, from the child’s point of view [26, 27]. It has 12 “care” and 13 “control” items each one being scored on a 4-point Likert scale. According to authors, cut-off scores were used to define a low level of Care for each parent, and a high level of Control. The Relationship Scales Questionnaire [28, 29] is a 30 items scale measuring on a 5-point Likert scale, two main dimensions of attachment: anxiety and avoidance. Avoidant attachment corresponds to individuals’ tendencies to withdraw from close relationships to regulate attachment-related behaviors and thoughts. Individuals high on this dimension are unwilling to rely on others [30]. Anxious attachment refers to the extent to which subjects are vigilantly attuned to attachment-relevant concerns, and worry about the availability and responsiveness of significant others. Attachment security can be defined as the absence of avoidance and anxiety [31, 32]. Convergent and discriminant validity of RSQ, as well as construct validity of anxious and avoidant dimensions have been demonstrated [28, 33].

### Statistical analysis

Independent t tests and chi-square tests were used to investigate differences in the FER scores, attachment dimensions, as well as clinical variables between the two groups. Within adverse events, two scores were constructed including the binary subscales of the CTQ (presence/absence) [25] and of the PBI (high/low level) [27]:

- a 4 points score of ‘disengaged environment’ by summing: 1. CTQ emotional neglect, 2. CTQ physical neglect, 3. PBI low level of care from mother, 4. PBI low level of care from father (each experience as 1 point, min = 0, max = 4)
- a 5 points score of ‘controlling environment’ including: 1. CTQ emotional abuse, 2. CTQ physical abuse, 3. CTQ sexual abuse, 4. PBI high level of control from mother, 5. PBI high level of control from father (each experience as 1 point, min = 0, max = 5).

These two scores were then correlated to FER, as well as to attachment dimensions. Two multiple linear regression analyses were conducted to investigate the predictive value of disengaged and controlling dimensions on FER scores, sensitivity and accuracy. In each of the regression analyses, group status (BPD versus control) was included, as well as the interaction between group status and the respective predictors. In a second time, attachment dimensions of anxiety and avoidance were added as covariables to the models and conducted to two supplementary analyses. In order to control the effect of psychotropic medication on FER, the use of psychotropic medication has been added as a covariable to the multiple linear regression analysis investigating the predictive value of disengaged and controlling dimensions on FER scores (see “Appendix”).

## Results

The patient group included 67.1% of inpatients, and 95.6% were currently under psychotropic medication. None of the control participants reported any current psychotropic medication use. SIPD score was 17.65 (3.91) for BPD patients and 2.12 (2.54) for HC.

The patient group included 67.1% of inpatients and 32.9% of outpatients, and 95.6% patients were currently under psychotropic medication. All subjects from the BPD group had at least one axis-I disorder. Mood disorders and anxiety were the most frequently observed comorbidities (respectively, 63.6% and 63.5%) followed by eating disorders (36.3%), post traumatic stress disorder (19.8%), disruptive behavior disorders (9.1%), and substance use disorders (9.1%). The most frequent personality disorder diagnosed in the BPD group was obsessive–compulsive (32%), followed by avoidant (14%), dependent (9%), antisocial (9%), and paranoid (4.5%).

As expected, adversity events occurred more frequently in BPD patients, compared to HC (Disengaged score _BPD_ = 2.4 (SD = 1.3), Disengaged score _HC_ = 0.9 (SD = 0.9); p < 0.001; Controlling score _BPD_ = 2.7 (SD = 1.4), Controlling score _HC_ = 0.9 (SD = 1); p < 0.001). Similarly, BPD patients had significantly higher scores on both attachment anxiety and attachment avoidance than matched controls (Anxiety score _BPD_ = -7.4 (SD = 6.4), Anxiety score _HC_ = -1.6 (SD = 6.4); p < 0.001; Avoidance score _BPD_ = -6.7 (SD = 9.8), Avoidance score _HC_ = -2.6 (SD = 6.7); p < 0.01). Comparison of FER skills showed that sensitivity was lower in BPD (N _BPD_ = 7.5 (SD = 5.5)) than in HC (N _HC_ = 11.4 (SD = 5.2); p < 0.001), whereas success rates were not statistically different between both (Success rate _BPD_ = 0.81 (SD = 0.1), Success rate _HC_ = 0.83 (SD = 0.1); p = 0.16).

**Fig. 2:**
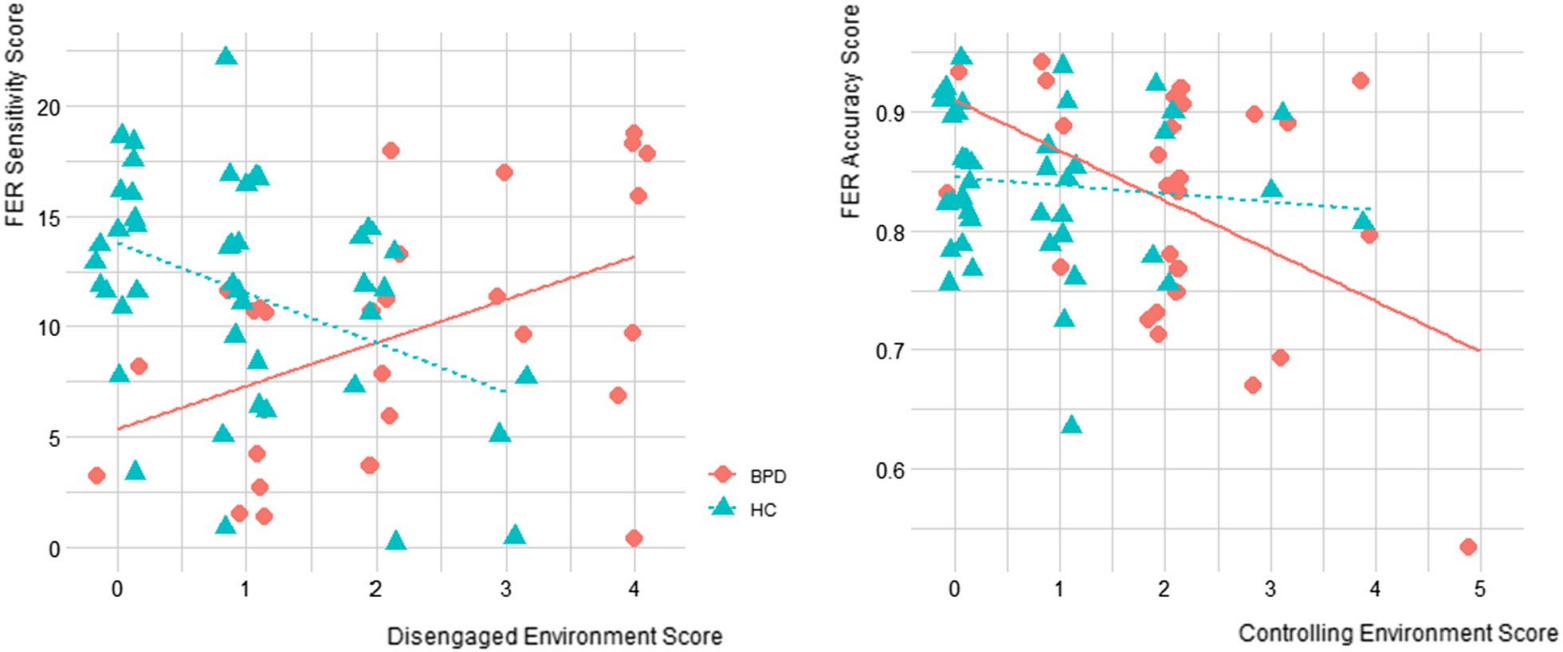
Correlations between Disengaged Environment and Sensitivity in Borderline Personality Disorders (BPD) and Healthy controls (HC)/ Controlling Environment and Accuracy in Borderline Personality Disorders (BPD) and Healthy controls (HC) (n = 85)

The linear correlations between dimensions of adversity, attachment and FER showed that disengaged environment was significantly negatively correlated with sensitivity in HC group, but significantly positively correlated with sensitivity in BPD group. The controlling environment was negatively associated with accuracy in BPD and in total sample. The linear correlations between adversity and attachment revealed that disengaged environment was positively and significantly correlated to anxious attachment in HC group and total sample, and significantly positively associated to avoidant attachment in total sample. The dimension of controlling environment was positively and significantly correlated to anxious attachment in HC group and in total sample (see Table 1, Fig. 2).

**Table 1:** Correlations between adversity, Facial emotion recognition (FER) and attachment in Borderline Personality Disorders (BPD), Healthy controls (HC) and whole sample (n = 85)

	Pearson’s correlations		
	whole sample	HC group	BPD group
	(N = 89)	(N = 44)	(N = 45)
*Disengaged environment x*			
FER Accuracy	0.01	0.19	− 0.02
FER Sensitivity	− 0.07	− 0.41 **	0.47*
Avoidant Attachment	0.25 **	0.07	0.17
Anxious Attachment	0.32 ***	0.22 *	0.07
*Controlling environment x*			
FER Accuracy	− 0.30 **	− 0.10	− 0.47*
FER Sensitivity	− 0.14	− 0.06	− 0.01
Avoidant Attachment	0.09	− 0.05	− 0.11
Anxious Attachment	0.37 ***	0.26*	0.12

*< .05, **< .01, ***< .001

As displayed in Table 2, there was an interaction between group status and disengaged environment for FER sensitivity. This interaction indicates that disengaged environment was associated with lower sensitivity in HC group, and with higher sensitivity in BPD group. There was also an interaction between group status and controlling environment for FER accuracy. This interaction indicates that controlling environment was associated with lower accuracy, but this effect was observed only in BPD group (See Fig. 2).

**Table 2:** Regression Analysis of Adversity Scores (Disengaged and Controlling Environment) and Group Status on Facial Emotion Recognition (FER) (n = 85)

	β	*t*	*p*	R^2^
*Accuracy*				
Group	− .02	− .93	.355	0.268
Disengaged environment	.01	1.17	.247	
Controlling environment	− .02	− 2.57	**.013**	
*Accuracy*				
Group	.07	1.79	.078	0.358
Disengaged environment	.02	1.50	.140	
Controlling environment	− .01	− .60	.548	
Group x Disengaged environment	− .02	− 1.36	.180	
Group x Controlling environment	− .04	− 2.25	**.028**	
*Sensitivity*				
Group	− 3.28	− 2.00	**.050**	0.209
Disengaged environment	.10	.19	.853	
Controlling environment	.12	.20	.846	
*Sensitivity*				
Group	− 8.93	− 3.42	**.001**	0.374
Disengaged environment	− 2.49	− 2.98	**.004**	
Controlling environment	.92	1.19	.240	
Group x Disengaged environment	4.25	3.98	**< .001**	
Group x Controlling environment	− .66	− .60	.552	

All models controlled by age, sex, CSP

Bold indicates threshold is significant, i.e. < 0.05

The regression analysis of adversity scores and attachment on FER revealed no specific effect of anxious or avoidant on FER, and no modification of previous predictions of environmental dimensions on FER (Table 3).

**Table 3:** Regression Analysis of Adversity Scores (Disengaged and Controlling Environment), Attachment and Group Status on Facial Emotion Recognition (FER) (n = 85)

	β	*t*	*p*	R^2^
*Accuracy*				
Group	0.08	1.88	.065	0.370
Disengaged environment	0.02	1.44	.155	
Controlling environment	− 0.01	− 0.78	.439	
Group x Disengaged environment	− 0.02	− 1.33	.189	
Group x Controlling environment	− 0.04	− 2.32	**.024**	
Avoidant Attachment	0.00	0.41	.682	
Anxious Attachment	− 0.00	− 0.77	.442	
*Sensitivity*				
Group	− 8.37	− 3.04	**.004**	0.362
Disengaged environment	− 2.38	− 2.77	**.008**	
Controlling environment	.84	1.06	.295	
Group x Disengaged environment	4.32	3.98	**< .001**	
Group x Controlling environment	− .90	− .79	.433	
Avoidant Attachment	.09	1.08	.286	
Anxious Attachment	− .03	− .27	.792	

All models controlled by age, sex, CSP

Bold indicates threshold is significant, i.e. < 0.05

The multiple linear regression including medication status in the predictive value of disengaged and controlling dimensions on FER scores revealed no significant effect of medication use (see “Appendix”).

## Discussion

In this study, we investigated whether two separate dimensions of adversity, disengaged and controlling environment, were associated with FER skills. Then we explored the role of attachment into the links between adversity and FER. First, our results showed that BPD patients’ score of accuracy was not different of HC’s, whereas sensitivity was lower in BPD, compared with HC. All levels of FER skills (including accuracy and sensitivity) of BPD patients compared to HC have been described among mixed results of studies in this field, from misidentification or enhanced FER in adult and adolescent patients [2, 4, 9]. Beyond the differences also related to the types of experimental paradigms, the most stable findings in literature data has pointed to enhanced vigilance leading to over-identification of social threat, which often (but not systematically), implies impairment in the evaluation of ambiguous facial expressions with a negative bias in emotion recognition [3, 4]. Patients in our study effectively needed more time than HC to correctly identify emotions when gradually increasing.

### Disengaged and controlling environment

Our study was aimed to explore what sort of social threat may influence FER. The results suggested distinct effects of environment on FER skills. The separation of adversity experiences into a bi-dimensional basis of deficit (withdrawal, neglect) or excess (control, abuse) of relational stimuli revealed that FER is differently influenced by these two types of factors, with a disengaged environment predicting a higher sensitivity and a controlling environment predicting a lower accuracy in BPD. It supports our first hypothesis that each adversity dimension would be correlated with FER through its own effect. It is also consistent with the findings that childhood trauma may have diverse impacts on social cognition and mentalization [2, 5, 10]. Our results reinforce these findings, in highlighting that the nature of childhood trauma itself is a key factor of influence of emotion perception.

These results support hypotheses that beyond the effects of abuse (much more often described in the studies on maltreatment than neglect), psychological neglect generates its own effects on psychological development and social cognitions [11, 12, 14]. In particular, maternal withdrawal over the first eight years of life has been described as associated with child caregiving behavior and with BPD in a high-risk longitudinal cohort from birth to 20 years old [13]. The caregiving attitude of children, often favored by parental disease, implies the development of vigilance and good perceptive skills, in order to protect the worrying parent. Our results suggest that the disengaged dimension of parent–child relationship (lack of responsiveness to the child’s affective needs, cold attitudes) may participate to enhance sensitivity in BPD. It seems that these subjects have put in place particular strategies of vigilance in the experiment, as, we can assume, towards the Caregiver(s). These links are in favor of specific modifications responding to the developmental context of the child exposed to relational withdrawal. It echoes Fonagy's model, which postulates that the impairment of mentalizing abilities in borderline subjects is associated with both traumatic experiences and a failure of parental mirroring, i.e., a failure of parents to put into words, to translate the child's affective states [34]. The results of our study suggest that the parental mirroring defect included in parental withdrawal attitudes would impact perceptual neural functioning at the emotional level. Thus, the disengaged and controlling types of parenting appear to be considered in terms of distinct traumatic pathway in borderline psychopathology, which may act simultaneously. Some correlations that have been observed previously between severity of maltreatment and FER in BPD patients led to envisage hyper- and hyposensitivity to emotions as an attempt of BPD patients to search for early signs of environmental threat, or contrarily as a protective mentalizing inhibition when trauma is severe [2, 6, 8, 9]. Our results highlight that it is not only the intensity of the trauma that influences emotional perception but its nature itself.

These distinct effects of adversity dimensions we observed in our study are also to consider with regards of the biosocial model of BPD [35]. Indeed, borderline pathology occurs when developmental vulnerability in childhood meets an invalidating environment that punishes and trivializes the child for his feelings. The concept of invalidating environment has been discussed in the light of the distinction between active emotional abuse and parental withdrawal, in which the child is not insulted or diminished, but his needs of nurturance and emotional comfort [12–14]. The distinction that our results bring about adversity dimensions, if confirmed, may shed light on confusing effects found with regard to Linehan’s hypothesis in studies about FER and mentalization in BPD, because these studies do not differentiate these different relational modalities. Furthermore, the inclusion of parental control dimension of PBI in our controlling dimension suggests that it is not only severity of trauma, which play a key role on FER, but the coercitive nature of a control stance in parental education or in abuse, along a continuous axis. Through the Control score, the PBI captures parental attitudes of invalidation, like discouraging the child of making his own decisions, invading his privacy, or making him feel dependent. A controlling style of parenting is known to decrease children self-esteem and to impair the development of a solid sense of self in childhood and adolescence [36], and this study may underline the perceptive psychopathological dimension of this impairment in the construction of the self.

### The place of attachment

The models including attachment dimensions did not elicit specific effect of attachment anxiety nor attachment avoidance on FER skills. Yet, studies described that disorganized attachment pattern may influence mentalizing in BPD [1]. For example, in a sample of borderline adolescents, a meditational effect of hypermentalizing has been identified between attachment and BPD features [37]. Anxiety attachment has been supposed to underlie hypersensitivity to emotions to others, because of their fear of abandon, and avoidant attachment has contrarily been supposed to underlie lower detection of emotional cues in their environment because of their tendency to deactivate attachment needs [15]. However, most of studies about FER and attachment did not include adverse events, and the articulation between adversity and attachment dimensions still remains unclear [9]. Thus, our results, if confirmed in future studies, suggest that attachment effects on FER skills may be explained by the nature of adversity in childhood. It confirmed our second hypothesis that the links between attachment and FER depended on adversity dimensions. The linear correlations highlighted a statistical link between disengaged environment and anxious, as well as avoidant attachment, whereas the dimension of controlling environment was preferentially linked to anxious attachment. While the literature has clearly shown a link between child maltreatment and attachment disorder [38–40], the links between different types of maltreatment and different attachment styles are more rarely explored. Oshri et al. [41] still found that sexual and emotional abuse were positively correlated with anxious and avoidant attachment styles in adolescents. Thus, our results encourage deepening these pathways from adversity to attachment in futures studies including larger samples.

### Attachment and trauma

The observation of different or opposite results between control and borderline groups, suggest that the effects of adversity are not the same at different doses or in different configurations. It echoes Linehan’s biosocial developmental model, which postulated that emotional dysregulation of BPD patients is characterized by a heightened sensitivity to emotions. In these patients, the regulation of stressful relational situations is overwhelmed, and not comparable to HC: it implies specific stress regulation modalities, including hypersensitivity to emotions and a slow return of stress level to baseline [6, 35]. Our results suggest distinct effects of trauma, including different regulation processes occurring in controls and in patients. In a previous study on RMET (Read the Mind in the Eyes Task) in BPD, avoidant attachment predicted lower emotion recognition in HC but not in BPD patients, and authors asked for exploring this surprising result [9]. In our study, sensitivity in HC decreased with the effect of disengaged environment, and increase in BPD. These results suggest that the prediction of lower FER by avoidant attachment in other studies is a facet of the link we described between disengaged environment and lower sensitivity in HC. The linear correlation between disengaged environment and avoidant attachment in our result supports also this hypothesis. These data are also consistent with the psychopathological model of BPD proposed by Fonagy and Luyten [34], which distinguishes distal causes (early caregiving, maltreatment) and proximal causes (sensitivity to stress, attachment) for the genesis of symptoms, including specific interactions at each level. Our results suggest that distal factors, represented by adversity events, play their own effects, independently of attachment (almost in HC, at low levels), and then, in a second level, interactions are of a different, and traumatic, nature in BPD.

### Cognitive processes of emotional stimuli

The distinction between sensitivity and accuracy in our experimental paradigm raises the question of different cognitive processes of emotional stimuli. Neuroimaging studies envisaged that faster engagement toward emotional stimuli indicates hypervigilance and failure to inhibit attention from these stimuli [42]. This exogenous orienting mode is associated to sub-cortical neural mechanisms or bottom-up network, whereas endogenous orienting is characterized as a top-down, voluntary attentional mode (and is associated with the anterior cingulate and prefrontal cortex). Some links have been made between Heart Rate Variability dysfunction (low resting HRV) and hypervigilance to emotional stimuli [43] in some anxious individuals who engage rapidly their attentional resources to identify emotions and are slower to disengage these resources on a second time. This attentional modality has been related to maladaptative emotion regulation, and evokes all the more emotional dysregulation of BPD patients. It would therefore be interesting to establish bridges between attentional orienting mode and the nature of childhood trauma.

### Limitations and perspectives

Some shortcoming of our study must be taken into consideration when interpreting these findings. The data are issued from an exploratory procedure (dividing adversity in two concepts), and the size of our sample lead us to temper the conclusions we may draw from this study. Besides, the assessment of child adversity, parenting and attachment was performed by self-reports questionnaires, which incorporates the possibility of a memory bias—more frequent in maltreatment histories—even if the CTQ has demonstrated good psychometric properties in international research [24, 25]. Moreover, effects of age, sex, socio-economical status (SES) were introduced in our models for more quality but the very limited number of boys, the small age range and the distribution of the SES make the analysis of these three variables unreliable in this study. Fourth, effect of medication use has been tested and statistically ruled out in this study, but medication concerned almost all patients and no healthy control subject. This imbalance puts the interpretation of the analysis into perspective. Among previous research on FER, results of the few studies that have looked at the impact of current medication on emotion recognition are very heterogeneous, and most of them found no difference between medicated and unmedicated groups [10, 44–47]. But to our knowledge, none of them has tested the effect of medication as a covariate in the relationship between adversity and emotion recognition. Thus, our results provide a basis for comparison in future studies.

## Conclusions

Despite these limitations, future perspectives may be drawn in the light of our study.

By including young adolescents, the research allows us to explore the mechanisms of trauma at an early stage of the disease. The present study supports clinical and theoretical observations suggesting that emotional sensitivity in BPD individuals is a core feature of the disorder from the beginning, and adds to the understanding of environmental dimensions, disengaged and controlling, as factors determining variations in sensitivity and accuracy. Finally, these results totally reinforce the recent advance in theories of cognition, which are switching from a solitary model of human perception to an altercentric nature of human cognition, in which emotion perception is strongly determined by social environment [48].

## Data Availability

https://figshare.com/articles/dataset/Adversity_and_REF_in_BPD/14605539/1. https://doi.org/10.6084/m9.figshare.14605539.v1, reference number [14605539].

## Appendix: Regression Analysis of Adversity Scores (Disengaged and Controlling Environment), Medication and Group Status on Facial Emotion Recognition (FER) (n = 85)

**Table:**

	β	*t*	*p*	R^2^
*Accuracy*				
Group	.09	1.97	.054	0.366
Disengaged environment	.02	1.49	.142	
Controlling environment	− .01	− .61	.546	
Psychotropic treatment	− .02	− .83	.409	
Group x Disengaged environment	− .03	− 1.49	.142	
Group x Controlling environment	− .04	− 2.15	.036	
*Sensitivity*				
Group	− 6.89	2.32	.024	0.395
Disengaged environment	− 2.50	3.02	.004	
Controlling environment	.92	− 1.19	.239	
Psychotropic treatment	− 2.69	1.40	.166	
Group x Disengaged environment	3.96	− 3.67	.001	
Group x Controlling environment	− .51	.46	.647	

## Abbreviations

BPD: Borderline personality disorders
CTQ: Childhood Trauma Questionnaire
FER: Facial emotion recognition
HC: Healthy control
PBI: Parental Bonding Instrument
RSQ: Relationship Scales Questionnaire
SD: Standard deviation
SES: Socio economical status
SIPD: Structural Interview for Personality Disorders

## References

[1] Fonagy P, Luyten P, Cicchetti D (2016). A multilevel perspective on the development of borderline personality disorder. Developmental psychopathology. Vol. 3: risk, disorder, and adaptation.

[2] Frick C, Lang S, Kotchoubey B, Sieswerda S, Dinu-Biringer R, Berger M, Veser S, Essig M, Barnow S (2012). Hypersensitivity in borderline personality disorder during mindreading. PLoS ONE.

[3] Berenson KR, Dochat C, Martin CG, Yang X, Rafaeli E, Downey G (2018). Identification of mental states and interpersonal functioning in borderline personality disorder. Personal Disord.

[4] Vestergaard M, Kongerslev MT, Thomsen MS, Mathiesen BB, Harmer CJ, Simonsen E, Miskowiak KW (2020). Women with borderline personality disorder show reduced identification of emotional facial expressions and a heightened negativity bias. J Pers Disord.

[5] Pears KC, Fisher PA (2005). Emotion understanding and theory of mind among maltreated children in foster care: evidence of deficits. Dev Psychopathol.

[6] Crowell SE, Beauchaine TP, Linehan MM (2009). A biosocial developmental model of borderline personality: elaborating and extending Linehan's theory. Psychol Bull.

[7] Porter C, Palmier-Claus J, Branitsky A, Mansell W, Warwick H, Varese F (2020). Childhood adversity and borderline personality disorder: a meta-analysis. Acta Psychiatr Scand.

[8] Nicol K, Pope M, Hall J (2014). Facial emotion recognition in borderline personality: an association, with childhood experience. Psychiatry Res.

[9] Van Heel M, Luyten P, De Meulemeester C, Vanwalleghem D, Vermote R, Lowyck B (2019). Mentalizing based on external features in borderline personality disorder compared with healthy controls: the role of attachment dimensions and childhood trauma. J Pers Disord.

[10] Lowyck B, Luyten P, Vanwalleghem D, Vermote R, Mayes LC, Crowley MJ (2016). What's in a face? Mentalizing in borderline personality disorder based on dynamically changing facial expressions. Personal Disord.

[11] Bowlby J (1988). A secure base: parent-child attachment and healthy human development.

[12] Schuder MR, Lyons-Ruth K, Osofsky JD (2004). "Hidden trauma" in infancy: attachment, fearful arousal, and early dysfunction of the stress response system. Young children and trauma: intervention and treatment.

[13] Lyons-Ruth K, Bureau JF, Easterbrooks MA, Obsuth I, Hennighausen K, Vulliez-Coady L (2013). Parsing the construct of maternal insensitivity: distinct longitudinal pathways associated with early maternal withdrawal. Attach Hum Dev.

[14] Briere J, Runtz M, Eadie E, Bigras N, Godbout N (2017). Disengaged parenting: Structural equation modeling with child abuse, insecure attachment, and adult symptomatology. Child Abuse Negl.

[15] Mikulincer M, Shaver PR, Cassidy J, Shaver PR (2008). Adult attachment and affect regulation. Handbook of attachment: theory, research, and clinical applications.

[16] Becker DF, Grilo CM, Edell WS, McGlashan TH (2002). Diagnostic efficiency of borderline personality disorder criteria in hospitalized adolescents: comparison with hospitalized adults. Am J Psychiatry.

[17] Chanen AM, McCutcheon L (2013). Prevention and early intervention for borderline personality disorder: current status and recent evidence. Br J Psychiatry Suppl.

[18] Anda RF, Felitti VJ, Bremner JD, Walker JD, Whitfield C, Perry BD, Dube SR, Giles WH (2006). The enduring effects of abuse and related adverse experiences in childhood. A convergence of evidence from neurobiology and epidemiology. Eur Arch Psychiatry Clin Neurosci.

[19] Kumari V (2020). Emotional abuse and neglect: time to focus on prevention and mental health consequences. Br J Psychiatry.

[20] Corcos M, Pham-Scottez A, Speranza M (2013). Le trouble de la Personnalité Borderline à l’adolescence.

[21] Pfohl B, Blum N, Zimmerman M (1997). Structured interview for DSM-IV personality (SIDP-IV).

[22] Ekman P, Friesen WV, 1976. Pictures of facial affect. Available from http://www.pauleckman.com/research_cds.php

[23] Blair RJ, Colledge E, Murray L, Mitchell DG (2001). A selective impairment in the processing of sad and fearful expressions in children with psychopathic tendencies. J Abnorm Child Psychol.

[24] Bernstein DP, Stein JA, Newcomb MD, Walker E, Pogge D, Ahluvalia T, Stokes J, Handelsman L, Medrano M, Desmond D, Zule W (2003). Development and validation of a brief screening version of the Childhood Trauma Questionnaire. Child Abuse Negl.

[25] Paquette D, Laporte L, Bigras M, Zoccolillo M (2004). Validation de la version française du CTQ et prévalence de l'histoire de maltraitance [Validation of the French version of the CTQ and prevalence of the history of maltreatment]. Sante Ment Que..

[26] Parker G (1989). The Parental Bonding Instrument: psychometric properties reviewed. Psychiatr Dev.

[27] Parker G, Tupling H, Brown LB (1979). A Parental Bonding Instrument. Br J Med Psychol.

[28] Griffin D, Bartholomew K (1994). Models of the self and other: Fundamental dimensions underlying measures of adult attachment. J Pers Soc Psychol.

[29] Bartholomew K, Horowitz LM (1991). Attachment styles among young adults: A test of a four-category model. J Pers Soc Psychol.

[30] Mikulincer M, Shaver PR, Zanna MP (2003). The attachment behavioral system in adulthood: activation, psychodynamics, and interpersonal processes. Advances in experimental social psychology.

[31] Brennan KA, Clark CL, Shaver PR, Simpson JA, Rholes WS (1998). Self-report measurement of adult attachment: an integrative overview. Attachment theory and close relationships.

[32] Suslow T, Dannlowski U, Arolt V, Ohrmann P (2010). Adult attachment avoidance and automatic affective response to sad facial expressions. Aust J Psychol.

[33] Bartholomew K (1990). Avoidance of intimacy: an attachment perspective. J Soc Pers Relat.

[34] Fonagy P, Luyten P (2009). A developmental, mentalization-based approach to the understanding and treatment of borderline personality disorder. Dev Psychopathol.

[35] Linehan MM (1995). Understanding borderline personality disorder.

[36] Soenens B, Vansteenkiste M, Luyten P, Duriez B, Goossens L (2005). Maladaptive perfectionistic self-representations: the mediational link between psychological control and adjustment. Pers Individ Differ.

[37] Sharp C, Venta A, Vanwoerden S, Schramm A, Ha C, Newlin E, Reddy R, Fonagy P (2016). First empirical evaluation of the link between attachment, social cognition and borderline features in adolescents. Compr Psychiatry.

[38] Bifulco A, Kwon J, Jacobs C, Moran PM, Bunn A, Beer N (2006). Adult attachment style as mediator between childhood neglect/abuse and adult depression and anxiety. Soc Psychiatry Psychiatr Epidemiol.

[39] Toth SL, Gravener-Davis JA, Guild DJ, Cicchetti D (2013). Relational interventions for child maltreatment: past, present, and future perspectives. Dev Psychopathol.

[40] Cicchetti D & Banny A. A developmental psychopathology perspective on child maltreatment. In: M. Lewis & K. D. Rudolph (Eds.), Handbook of developmental psychopathology, 2014, pp. 723–41.

[41] Oshri A, Sutton TE, Clay-Warner J, Miller JD (2015). Child maltreatment types and risk behaviors: associations with attachment style and emotion regulation dimensions. Pers Individ Differ.

[42] Sussman TJ, Jin J, Mohanty A (2016). Top-down and bottom-up factors in threat-related perception and attention in anxiety. Biol Psychol.

[43] Park G, Thayer JF (2014). From the heart to the mind: cardiac vagal tone modulates top-down and bottom-up visual perception and attention to emotional stimuli. Front Psychol.

[44] Lynch TR, Rosenthal MZ, Kosson DS, Cheavens JS, Lejuez CW, Blair RJR (2006). Heightened sensitivity to facial expressions of emotion in borderline personality disorder. Emotion.

[45] Brotman MA, Guyer AE, Lawson ES, Horsey SE, Rich BA, Dickstein DP (2008). Facial emotion labeling deficits in children and adolescents at risk of Bipolar Disorder. Am J Psychiatry.

[46] Kalmar JH, Wang F, Chepenik LG, Womer FY, Jones MM, Pittman B (2009). Relation between amygdala structure and function in adolescents with bipolar disorder. J Am Acad Child Adolescent Psychiatry.

[47] Jänsch C, Harmer C, Cooper MJ (2009). Emotional processing in women with anorexia nervosa and in healthy volunteers. Eat Behav.

[48] Kampis D, Southgate V (2020). Altercentric cognition: how others influence our cognitive processing. Trends Cogn Sci.

